# Comparative Efficacy of Pharmacological Treatments for Adults With Autosomal Dominant Polycystic Kidney Disease: A Systematic Review and Network Meta-Analysis of Randomized Controlled Trials

**DOI:** 10.3389/fphar.2022.885457

**Published:** 2022-05-18

**Authors:** Shunichiro Tsukamoto, Shingo Urate, Takayuki Yamada, Kengo Azushima, Takahiro Yamaji, Sho Kinguchi, Kazushi Uneda, Tomohiko Kanaoka, Hiromichi Wakui, Kouichi Tamura

**Affiliations:** ^1^ Department of Medical Science and Cardiorenal Medicine, Yokohama City University Graduate School of Medicine, Yokohama, Japan; ^2^ Renal-Electrolyte Division, Department of Medicine, University of Pittsburgh, Pittsburgh, PA, United States; ^3^ Cardiovascular and Metabolic Disorders Program, Duke-NUS Medical School, Singapore, Singapore; ^4^ Department of Kampo Medicine, Aizu Medical Center, Fukushima Medical University School of Medicine, Aizuwakamatsu, Japan

**Keywords:** autosomal dominant polycystic kidney disease, tolvaptan, network meta-analysis, kidney function, total kidney volume

## Abstract

**Background:** Tolvaptan is the gold standard treatment for autosomal dominant polycystic kidney disease (ADPKD), while several other drugs have the potential to inhibit the progression of ADPKD. However, individual clinical trials may not show sufficient differences in clinical efficacy due to small sample sizes. Furthermore, the differences in therapeutic efficacy among drugs are unclear. Herein, we investigated the effect of the ADPKD treatments.

**Methods:** We systematically searched PubMed, Medline, EMBASE, and the Cochrane Library through January 2022 to identify randomized controlled trials in ADPKD patients that compared the effects of treatments with placebo or conventional therapy. A network meta-analysis was performed to compare the treatments indirectly. The primary outcomes were changes in kidney function and the rate of total kidney volume (TKV) growth.

**Results:** Sixteen studies were selected with a total of 4,391 patients. Tolvaptan significantly preserved kidney function and inhibited TKV growth compared to the placebo {standardized mean difference (SMD) [95% confidence interval (CI)]: 0.24 (0.16; 0.31) and MD: −2.70 (−3.10; −2.30), respectively}. Tyrosine kinase inhibitors and mammalian target of rapamycin (mTOR) inhibitors inhibited TKV growth compared to the placebo; somatostatin analogs significantly inhibited TKV growth compared to the placebo and tolvaptan [MD: −5.69 (−7.34; −4.03) and MD: −2.99 (−4.69; −1.29), respectively]. Metformin tended to preserve renal function, although it was not significant [SMD: 0.28 (−0.05; 0.61), *p* = 0.09].

**Conclusion:** The therapeutic effect of tolvaptan was reasonable as the gold standard for ADPKD treatment, while somatostatin analogs also showed notable efficacy in inhibiting TKV growth.

**Systematic Review Registration**: https://www.crd.york.ac.uk/prospero/, identifier CRD42022300814.

## 1 Introduction

Autosomal dominant polycystic kidney disease (ADPKD) is the most frequent congenital genetic disorder leading to renal failure, with an estimated prevalence of 1:400–1:1,000 (Chebib and Torres, 2016). ADPKD accounts for 5–10% of cases of end-stage renal disease and is the fourth leading cause of kidney failure worldwide (Chebib and Torres, 2016). Although ADPKD is considered to be a slowly progressing disease, once kidney volume reaches a critical size, the glomerular filtration rate (GFR) undergoes a sharp decline (Muto et al., 2021). The increase in kidney volume was reported to be an important predictor of kidney prognosis (Bergmann et al., 2018). In addition, increased kidney volume may impair quality of life in patients with ADPKD by causing kidney pain and abdominal distention (Bergmann et al., 2018). Therefore, not only the direct maintenance of kidney function but also preventing cyst formation and growth of kidney volume are treatment targets for ADPKD.

ADPKD is mainly caused by mutations in the PKD1 and PKD2 genes, which encode polycystin-1 (PC1) and polycystin-2 (PC2) (Capuano et al., 2022). Mutations in PCs are associated with decreased intracellular calcium levels and increased cyclic adenosine monophosphate (cAMP) production via increased adenylyl cyclase activity (Mahendran et al., 2021). Upregulation of cAMP leads to activation of protein kinase A, which promotes the formation of cysts, and chloride and fluid secretion, through the cystic fibrosis transmembrane conductance regulator (CFTR) (Hanaoka et al., 1996). Upregulation of cAMP also activated mitogen-activated protein kinase and mammalian target of rapamycin (mTOR) (Distefano et al., 2009; Spirli et al., 2010). Janus kinase and signal transducers and activators of transcription signaling are also involved in ADPKD (Fragiadaki et al., 2017; Mahendran et al., 2021). Furthermore, AMP-activated protein kinase (AMPK) downregulates CFTR channels and the mTOR pathway in ADPKD (Mahendran et al., 2021).

Several treatments have been investigated based on these molecular mechanisms. The current gold standard for ADPKD treatment is tolvaptan, a vasopressin-2 receptor (V2R) antagonist (Torres et al., 2012). However, the goal of completely inhibiting the progression of ADPKD has not been achieved. In addition, in some cases, the adverse effects specific to tolvaptan prevent adequate therapeutic efficacy (Torres et al., 2012). In some countries, somatostatin analogs, long-acting release octreotide (octreotide-LAR), have been approved for treatment (Capuano et al., 2022). Both of these drugs are targeted at lowering intracellular cAMP levels (Torres and Harris, 2014). Several drugs, such as mTOR inhibitors, which target activated mTOR signaling (Lin et al., 2019) or metformin and pravastatin, which target activation of AMPK (Capuano et al., 2022), are also promising agents to treat ADPKD. Although some studies have shown that such emerging agents are effective (Walz et al., 2010; Pisani et al., 2018), clinically sufficient differences in efficacy may not have been confirmed because of the small sample sizes of individual trials (Perrone et al., 2021; Brosnahan et al., 2022). In addition, no study has compared the effects of these drugs. Data regarding relative efficacy and adverse events (AEs) of each drug are informative for patients and physicians. Herein, we investigated the safety and efficacy of these drug treatments for ADPKD patients using a network meta-analysis.

## 2 Methods

### 2.1 Literature Search

The search strategy was conducted following the Preferred Reporting Items for Systematic Reviews and Meta-Analyses (PRISMA) extension statement for network meta-analysis (Moher et al., 2009; Hutton et al., 2015). The protocol is registered in the International Prospective Register of Systematic Reviews (PROSPERO) with identification number CRD42022300814.

We performed a systematic search of PubMed, Medline, EMBASE, and the Cochrane Library from inception to January 8, 2022. The following keywords were applied: (“polycystic kidney” [tiab] OR “polycystic kidney disease” [tiab] OR PKD [tiab] OR ADPKD [tiab] OR “autosomic dominant polycystic kidney disease” [tiab] OR “autosomal dominant polycystic kidney disease” [tiab] OR Polycystic Kidney, Autosomal Dominant [MeSH]) AND (randomized controlled trial [pt] OR controlled clinical trial [pt] OR randomized [tiab] OR placebo [tiab] OR clinical trials as topic [mesh: noexp] OR randomly [tiab] OR trial [ti]).

### 2.2 Study Selection

Studies were eligible for inclusion if the following criteria were met: published in a peer-reviewed journal; included adults (age ≥ 18 years) with a clinical diagnosis of ADPKD; randomized controlled trial (RCT) comparing metformin, somatostatin analogs, tyrosine kinase inhibitors (TKIs), niacinamide, mTOR inhibitors, tolvaptan, or statins with placebo or only conventional therapy (not receiving placebo); followed participants for at least 12 months post-randomization; and reported the annual change in kidney function or annual growth rate of total kidney volume (TKV) or height-adjusted TKV (htTKV). Studies were excluded if they were 1) crossover trials, 2) included dialysis patients and/or patients who underwent kidney transplantation, or 3) there were insufficient data for analysis even after contacting the authors. The reference lists of the studies included in the meta-analysis were reviewed to minimize missing relevant studies. Two independent authors (S.T. and S.U.) reviewed the search results separately and in a blinded manner to select studies based on the inclusion and exclusion criteria. When a consensus was not reached between the two authors, a third author (T.Y.) was consulted to reach a decision.

### 2.3 Outcomes

The primary outcomes were the annual change in kidney function and the annual growth rate of TKV or htTKV. Kidney function was assessed using the measured GFR (mGFR) or estimated GFR (eGFR) (mL/min or mL/min/1.73 m^2^, respectively). Typical AEs for each drug were listed for safety. Statistical analysis was conducted for serious AEs, nausea/vomiting, diarrhea, urinary tract infection (UTI), and fatigue/weakness, which were described for most drugs. Subgroup analysis was performed according to age (≤ 65 years), baseline eGFR (≥ 30 ml/min/1.73 m^2^), and TKV (≥ 750 cc).

### 2.4 Data Extraction and Quality Assessment

All data from the eligible studies were extracted independently by two investigators (S.T. and S.U.). Any conflicts in data extraction or quality assessment were resolved by a third reviewer (T.Y.). In each study, we extracted data on the annual change in mGFR or eGFR, the annual growth rate of TKV or htTKV, and the incidence of AEs in each group. Some data were obtained by calculation. We used the Cochrane risk of bias assessment to explore the sources of bias in the RCTs included in the analysis (Higgins et al., 2011). Applying this tool, we evaluated the risk of bias during random sequence generation, allocation concealment, the blinding of participants and researchers, the blinding of the outcome assessments, selective reporting, incomplete outcome data, and other metrics. Funnel plot asymmetry tests, the Egger’s test, and the Begg-Mazumdar test were used to assess for potential evidence of reporting bias. Funnel plot asymmetry tests were only performed when there were at least ten studies (Sterne et al., 2011).

### 2.5 Statistical Analysis

The annual change in kidney function (mGFR or eGFR), which was a continuous value, was calculated with the standardized mean difference (SMD) and 95% confidence interval (CI) because the scale was not consistent between mGFR and eGFR. The annual growth rate of TKV or htTKV was calculated as the mean difference (MD) and 95% CI because TKV and htTKV had the same scale for the rate of change (%). The results of dichotomous outcomes, such as AEs, were estimated as risk ratios (RRs) and 95% CIs. We performed a network meta-analysis using the netmeta package (version 1.1-0) in R programming language (The R Foundation for Statistical Computing, Vienna, Austria). A random-effects model was used for analysis. Heterogeneity was assessed using the *p*-value of the I^2^ variable (Higgins and Thompson, 2002; Higgins et al., 2003). Heterogeneity was considered to be low, moderate, or high if I^2^ was 25%, 50%, or 75%, respectively. All *p*-values < 0.05 were considered significant.

## 3 Results

### 3.1 Literature Search and the Included Studies

**FIGURE 1:**
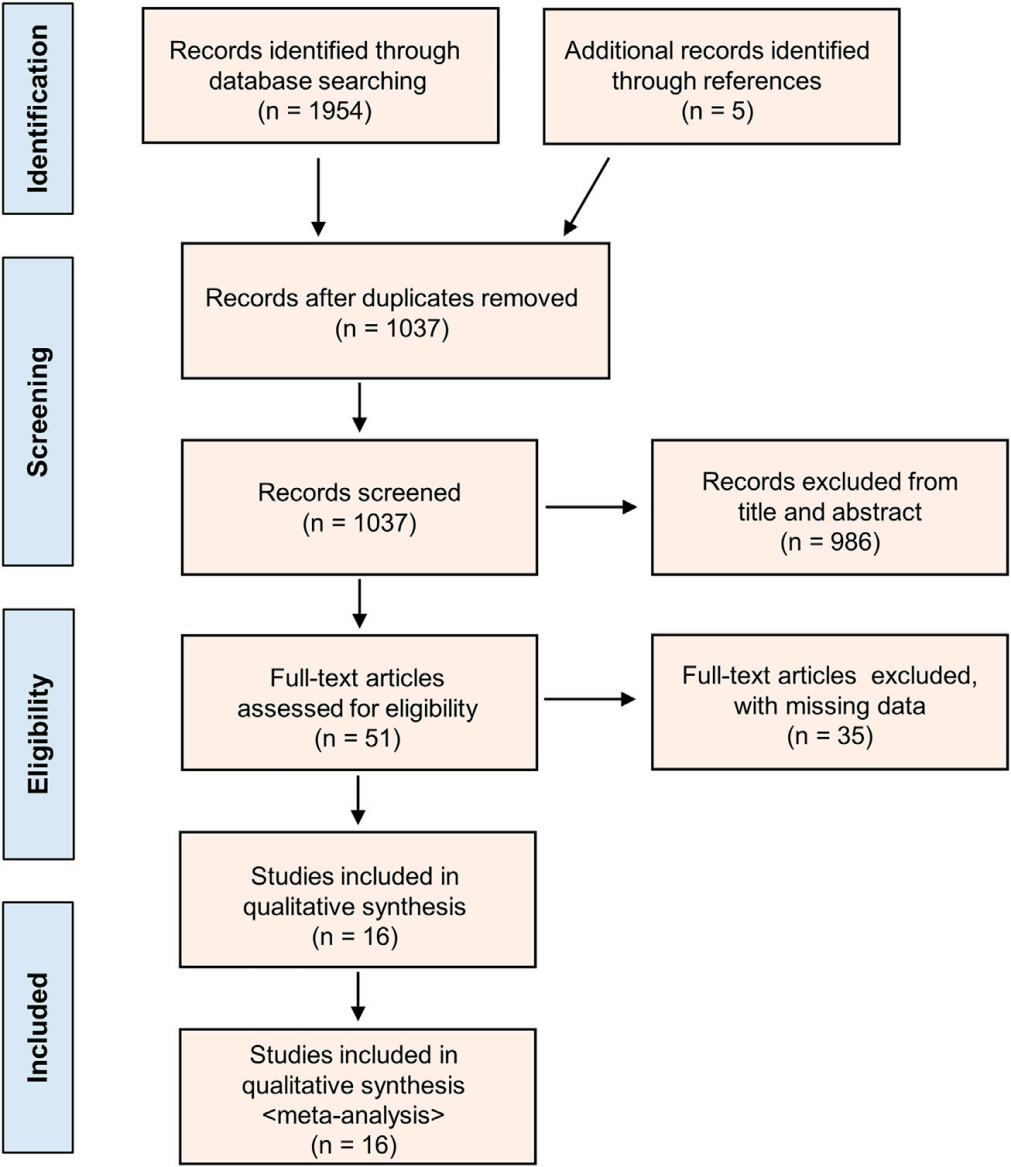
PRISMA flow diagram showing the study selection.

A diagram of the study selection is shown in Figure 1. A total of 1,954 studies were identified in the primary database search, and five additional studies were identified in the references. We removed 922 duplicate studies; thus, 1,037 studies were screened. By screening the titles and abstracts, 986 papers were excluded because they did not meet the inclusion criteria. Thirty-five additional studies were excluded after assessing the full-text articles due to missing data. Finally, sixteen studies published up to January 8, 2022, were selected for our meta-analysis according to the inclusion criteria (Fassett et al., 2010; Hogan et al., 2010; Serra et al., 2010; Walz et al., 2010; Torres et al., 2012; Caroli et al., 2013; Braun et al., 2014; Ruggenenti et al., 2016; Torres et al., 2017a; Tesar et al., 2017; Meijer et al., 2018; Perico et al., 2019; El Ters et al., 2020; Hogan et al., 2020; Perrone et al., 2021; Brosnahan et al., 2022).

Of the sixteen RCTs, two were on metformin, five were on somatostatin analogs, four were on mTOR inhibitors, two were on tolvaptan, and the rest were on TKIs, niacinamide, and statins, respectively. The pooled population consisted of 4,391 patients (75 treated with metformin, 305 treated with somatostatin analogs, 304 treated with mTOR inhibitors, 1,644 treated with tolvaptan, 113 treated with TKI, 18 treated with niacinamide, 29 treated with statin, 252 treated with conventional therapy, and 1,651 treated with placebo). Among the drug treatment groups, tolvaptan had the largest number of patients. Conventional therapy consisted of dietary advice and blood pressure control with antihypertensive drugs.

### 3.2 Study Characteristics and Quality Assessment

The characteristics of the included studies are shown in Table 1. Table 2 highlights the various baseline parameters of the individual studies. More detailed patient characteristics and the quality assessment of the included studies are shown in Supplementary Table S1; Supplementary Figure S1, respectively.

**TABLE 1:** The characteristics of the included studies.

Study			Intervention			Control		Outcomes (included in this analysis)
First author (Year)	Design	Follow-up Duration (Years)	Number of patients (% male)	Treatment drug (Group)	Treatment dose	Number of patients (% male)	Treatment	
Brosnahan et al. (2022)	RCT	1	26 (42)	Metformin (Metformin)	500–1,000 mg twice a day	25 (32)	Placebo	htTKV % change
								eGFR
								AEs
Perrone et al. (2021)	RCT	1	49 (NA)	Metformin (Metformin)	500 mg once a day to 1,000 mg twice a day	48 (NA)	Placebo	htTKV % change
								eGFR
								AEs
Hogan et al. (2020)	RCT	2	33 (6)	Pasireotide (Somatostatin analogue)	60 mg intramuscularly every 28 days	15 (20)	Placebo	htTKV % change
								eGFR
								AEs
El Ters et al. (2020)	RCT	1	18 (56)	Niacinamide (Niacinamide)	30 mg/kg/day	18 (33)	Placebo	htTKV % change
								AEs
Perico et al. (2019)	RCT	3	51 (61)	Octreotide (Somatostatin analogue)	20 mg intramuscularly every 28 days	49 (53)	Placebo	TKV % change (median % change) iohexol GFR
								AEs
Meijer et al. (2018)	RCT	5	153 (46.4)	Lanreotide (Somatostatin analogue)	60–120 mg subcutaneously every 4 weeks	152 (46.7)	Conventional therapy	htTKV % change
								eGFR
								AEs
Torres et al. (2017a)	RCT	1	683 (50.8)	Tolvaptan (Tolvaptan)	Twice daily (30–45 and 15 mg) orally	687 (48.5)	Placebo	eGFR
								AEs
Tesar et al. (2017)	RCT	2	113 (50)	Bosutinib (TKI)	200–400 mg/day	56 (37)	Placebo	TKV % change eGFR
								AEs
Ruggenenti et al. (2016)	RCT	1	21 (42.9)	Sirolimus (mTOR inhibitor)	2.2 ± 0.7 mg/day	20 (40)	Conventional therapy	TKV % change iohexol GFR
								AEs
Braun et al. (2014)	RCT	1	20 (50)	Sirolimus (mTOR inhibitor)	TBL adjusted 2–5 ng/ml or TBL > 5–8 ng/ml	10 (70)	Conventional therapy	TKV % change eGFR
								AEs
Caroli et al. (2013)	RCT	3	40 (42.5)	Octreotide (Somatostatin analogue)	20 mg intramuscularly twice every 28 days	39 (51.3)	Placebo	htTKV % change
								iohexol GFR
								AEs
Torres ete al., (2012)	RCT	3	961 (51.5)	Tolvaptan (Tolvaptan)	Twice daily (45–90 mg and 15–30 mg) orally	484 (51.9)	Placebo	TKV % change eGFR
								AEs
Walz et al. (2010)	RCT	2	213 (48.8)	Everolimus (mTOR inhibitor)	TBL adjusted 3–8 ng/ml	216 (53.7)	Placebo	TKV % change (median % change)
								AEs
Serra et al. (2010)	RCT	1.5	50 (58)	Sirolimus (mTOR inhibitor)	Steady state levels adjusted	50 (64)	Conventional therapy	AEs
					4–10 ng/ml			
Hogan et al. (2010)	RCT	1	28 (17.9)	Octreotide (Somatostatin analogue)	10–20 mg intramuscularly twice every 28 days	14 (7.1)	Placebo	TKV % change iothalamate GFR
								AEs
Fassett et al. (2010)	RCT	2	29 (41)	Pravastatin (Statin)	20 mg/day	20 (35)	Conventional therapy	eGFR

RCT, randomized controlled trial; TKV, total kidney volume; htTKV; height adjusted TKV; eGFR, estimated glomerular filtration rate; AE, adverse event; TKI, tyrosine kinase inhibitor.

**TABLE 2:** Baseline parameters of the individual studies.

First author (Year)	Treatment group	Age (year)	S-Cr (mg/dl)	eGFR (mL/min/1.73m^2^)	TKV (ml)	htTKV (ml/m)	BP (mmHg)	BMI (kg/m^2^)
Brosnahan et al. (2022)	Metformin	48	NA	68	2101	1281	124/80	29.2
	Placebo	48	NA	72	1156	688	125/82	28.4
Perrone et al. (2021)	Metformin	42	NA	86	NA	626	122/77	27.0
	Placebo	42	NA	86	NA	751	124/75	26.6
Hogan et al. (2020)	Pasireotide LAR	50	1.0	74	NA	534	NA	26.0
	Placebo	51	1.0	76	NA	397	NA	26.1
El Ters et al. (2020)	Niacinamide	40	NA	78	NA	1210	NA	NA
	Placebo	45	NA	68	NA	1021	NA	NA
Perico et al. (2019)	Octreotide LAR	49	229.8 μmol/L	27.9^a^	2338	1344	135/82	NA
	Placebo	50	238.7 μmol/L	25.8^a^	2591	1528	132/83	NA
Meijer et al. (2018)	Lanreotide	48	1.5	51	2046	1138	132/82	26.9
	Conventional therapy	49	1.5	51	1874	1029	133/82	27.1
Torres et al. (2017a)	Tolvaptan	47	NA	41	NA	NA	129/82	28.0
	Placebo	47	NA	41	NA	NA	130/83	27.7
Tesar et al. (2017)	Bosutinib	39	NA	87	1393	NA	NA	NA
	Placebo	39	NA	87	1392	NA	NA	NA
Ruggenenti et al. (2016)	Sirolimus	49	2.9	27^a^	2858	NA	136/86	NA
	Conventional therapy	48	2.5	31^a^	3123	NA	134/86	NA
Braun et al. (2014)	Sirolimus	49	NA	68	2099	NA	NA	NA
	Conventional therapy	49	NA	70	2072	NA	NA	NA
Caroli et al. (2013)	Octreotide LAR	36	92 μmol/L	90	1557	906	127/84	NA
	Placebo	38	108 μmol/L	76	2161	1267	127/84	NA
Torres ete al., (2012)	Tolvaptan	39	1.1	81	1705	979	129/83	NA
	Placebo	39	1.0	82	1668	958	128/83	NA
Walz et al. (2010)	Everolimus	45	1.4	53	2028	NA	136/88	25.7
	Placebo	44	1.4	56	1911	NA	135/88	26.0
Serra et al. (2010)	Sirolimus	31	NA	92	907	NA	130/84	25
	Conventional therapy	32	NA	91	1003	NA	130/83	24
Hogan et al. (2010)	Octreotide LAR	50	1.1	70^b^	1143	NA	122/80	26.3
	Placebo	50	1.1	71^b^	803	NA	121/79	24.4
Fassett et al. (2010)	Pravastatin	53	NA	59	NA	NA	133/88	NA
	Conventional therapy	49	NA	50	NA	NA	134/82	NA

S-Cr, Serum creatinine; eGFR, estimated glomerular filtration rate; TKV, total kidney volume; htTKV, height-adjusted TKV; BP, blood pressure; BMI, body mass index; LAR, long-acting release.

a Iohexol GFR.

b Iothalamate GFR.

### 3.3 Network Meta-Analysis of the Treatment Groups

#### 3.3.1 Kidney Function (GFR)

**FIGURE 2:**
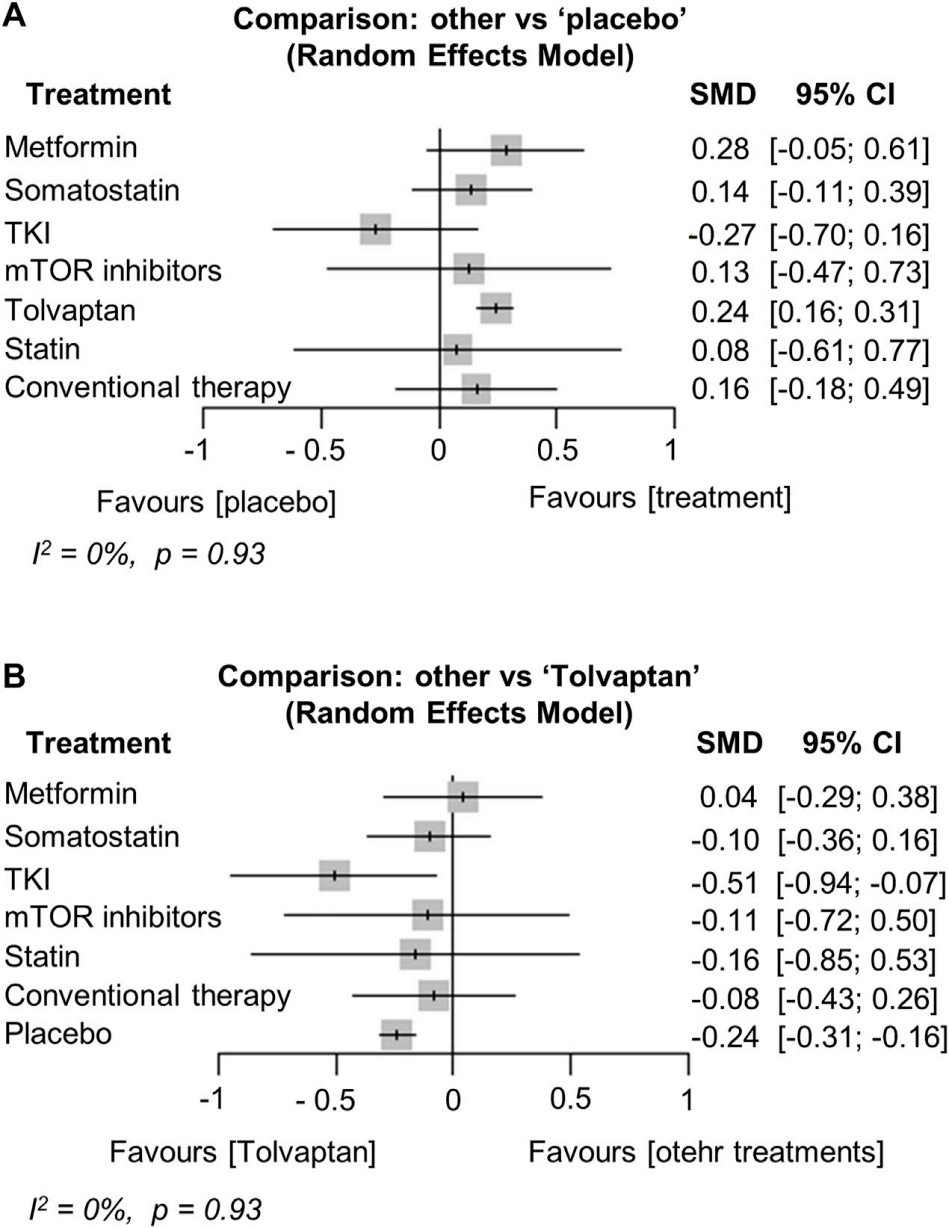
Network meta-analysis reporting the standard mean difference (SMD) for each treatment effect of preserving kidney function (glomerular filtration rate) compared to **(A)** the placebo and **(B)** tolvaptan in ADPKD patients. CI, confidence interval; TKI, tyrosine kinase inhibitor; ADPKD, autosomal dominant polycystic kidney disease.

Network plots are shown in Supplementary Data (Supplementary Figure S2). Kidney function was compared with the SMD in the annual change of mGFR or eGFR, and thirteen studies were included in the analysis (Fassett et al., 2010; Hogan et al., 2010; Torres et al., 2012; Caroli et al., 2013; Braun et al., 2014; Ruggenenti et al., 2016; Torres et al., 2017a; Tesar et al., 2017; Meijer et al., 2018; Perico et al., 2019; Hogan et al., 2020; Perrone et al., 2021; Brosnahan et al., 2022). The analysis showed that tolvaptan significantly preserved GFR compared to the placebo [SMD (95% CI) vs. placebo: 0.24 (0.16; 0.31), *p* < 0.001]. Metformin also tended to retain the GFR compared to the placebo [SMD (95% CI) vs. placebo: 0.28 (−0.05; 0.61), *p* = 0.09] (Figure 2A). The change in GFR did not differ from placebo after treatment with somatostatin analogs, mTOR inhibitors, and statins (Figure 2A). Moreover, the change in the GFR after the TKI treatment was not different from the placebo but was significantly worse than tolvaptan [SMD (95% CI) vs. tolvaptan: −0.51 (−0.94; −0.07), *p* = 0.02] (Figure 2B). Heterogeneity in this analysis was low (I^2^ = 0%, *p* = 0.93).

#### 3.3.2 Total Kidney Volume

**FIGURE 3:**
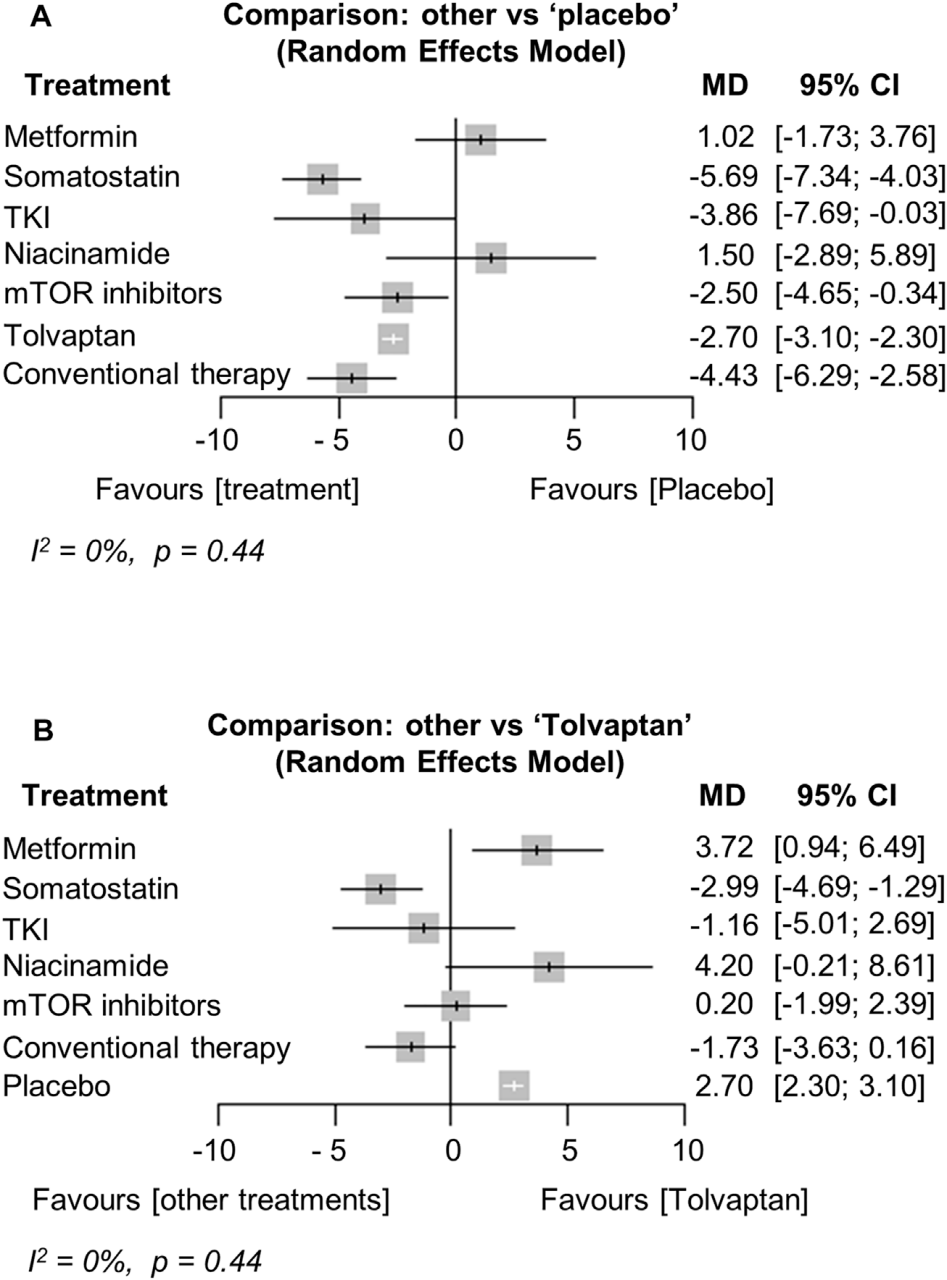
Network meta-analysis reporting the mean difference (MD) for each treatment effect of inhibiting total kidney volume (TKV) growth rate compared to **(A)** the placebo and **(B)** tolvaptan in ADPKD patients. CI, confidence interval; TKI, tyrosine kinase inhibitor; ADPKD, autosomal dominant polycystic kidney disease.

The network plots are shown in Supplementary Data (Supplementary Figure S3). Thirteen studies were included in the analysis of the annual growth rate of TKV (or htTKV) (Hogan et al., 2010; Walz et al., 2010; Torres et al., 2012; Caroli et al., 2013; Braun et al., 2014; Ruggenenti et al., 2016; Tesar et al., 2017; Meijer et al., 2018; Perico et al., 2019; El Ters et al., 2020; Hogan et al., 2020; Perrone et al., 2021; Brosnahan et al., 2022). The analysis showed that treatment with somatostatin analogs, TKIs, mTOR inhibitors, or tolvaptan significantly reduced the TKV growth rate compared to the placebo [MD (95% CI) vs. placebo: −5.69 (−7.34; −4.03), *p* < 0.001, −3.86 (−7.69; −0.03), *p* = 0.05, −2.50 (−4.65; −0.34), *p* = 0.02, −2.70 (−3.10; −2.30), *p* < 0.001, respectively] (Figure 3A). In particular, treatment with somatostatin analogs was the most effective at reducing the TKV growth rate, and the effect was significantly better than that of tolvaptan [MD (95% CI) vs. tolvaptan: −2.99 (−4.69; −1.29), *p* = 0.001] (Figure 3B). Interestingly, conventional therapy significantly suppressed TKV growth compared to the placebo [MD (95% CI) vs. placebo: −4.43 (−6.29; −2.58), *p* < 0.001] (Figure 3A). In contrast, the TKV growth rate of the metformin and niacinamide treatments did not differ from the placebo (Figure 3A). Heterogeneity in this analysis was low (I^2^ = 0%, *p* = 0.44).

#### 3.3.3 Adverse Events

**FIGURE 4:**
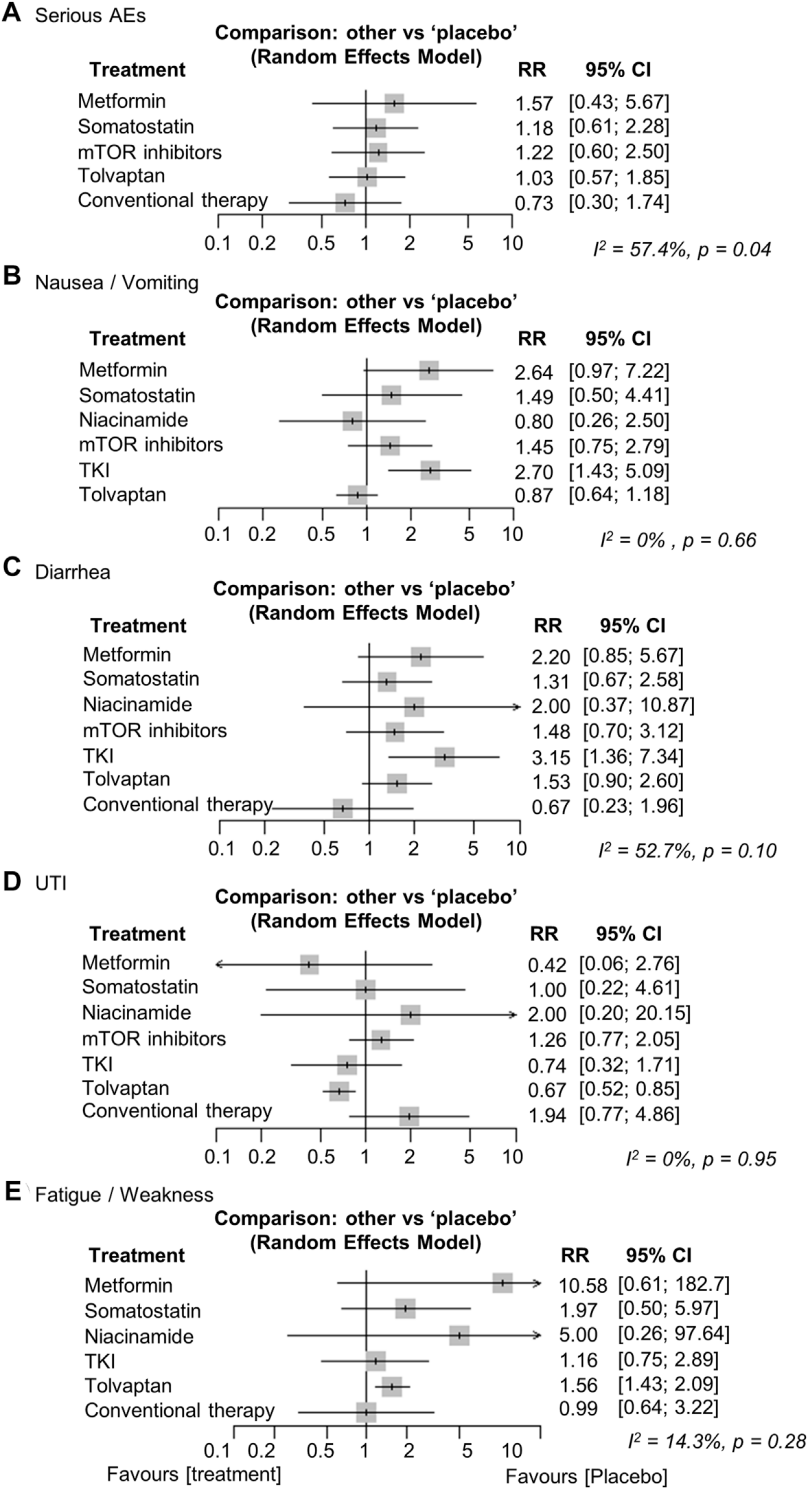
Network meta-analysis reporting the risk ratios (RRs) for adverse events (AEs) regarding **(A)** serious AEs, **(B)** nausea/vomiting, **(C)** diarrhea, **(D)** urinary tract infection (UTI), and **(E)** fatigue/weakness in ADPKD patients. CI, confidence interval; TKI, tyrosine kinase inhibitor; ADPKD, autosomal dominant polycystic kidney disease.

The major AEs differed for each treatment. Metformin and somatostatin analogs resulted in relatively more gastrointestinal AEs, such as nausea/vomiting and diarrhea (Hogan et al., 2010; Caroli et al., 2013; Meijer et al., 2018; Perico et al., 2019; Hogan et al., 2020; Perrone et al., 2021; Brosnahan et al., 2022). Aphthous stomatitis was more frequent in the mTOR inhibitor treatment group and edema and diarrhea were also observed (Serra et al., 2010; Walz et al., 2010; Braun et al., 2014; Ruggenenti et al., 2016). In the tolvaptan treatment group, polyuria, nocturia, thirst, and increases in liver enzymes were more common AEs (Torres et al., 2012; Torres et al., 2017b). We analyzed statistically the frequency of nausea/vomiting, diarrhea, UTI, and fatigue/weakness because they were observed in most of the treatment groups. Serious AEs were also analyzed based on the numbers listed in the articles. We did not analyze AEs that were not mentioned in the studies. No significant difference in the frequency of serious AEs was observed between the treatment groups and the placebo (heterogeneity, high, I^2^ = 57.4%, *p* = 0.04) (Walz et al., 2010; Torres et al., 2012; Caroli et al., 2013; Braun et al., 2014; Ruggenenti et al., 2016; Torres et al., 2017b; Meijer et al., 2018; Perico et al., 2019; Hogan et al., 2020; Perrone et al., 2021) (Figure 4A). Nausea/vomiting and diarrhea increased significantly in the TKI treatment group compared to the placebo [RR (95% CI): 2.70 (1.43; 5.09), *p* < 0.01, and 3.15 (1.36; 7.34), *p* < 0.01, respectively] (Figures 4B,C). UTI significantly decreased in the tolvaptan treatment group compared to the placebo [RR (95% CI): 0.67 (0.52; 0.85), *p* < 0.01] (Figure 4D). Fatigue/weakness increased significantly in the tolvaptan treatment group compared to the placebo [RR (95% CI): 1.56 (1.16; 2.09), *p* < 0.01] (Figure 4E). The network plots for each analysis are shown in Supplementary Data (Supplementary Figures S4A–E).

#### 3.3.4 Subgroup Analysis

**FIGURE 5:**
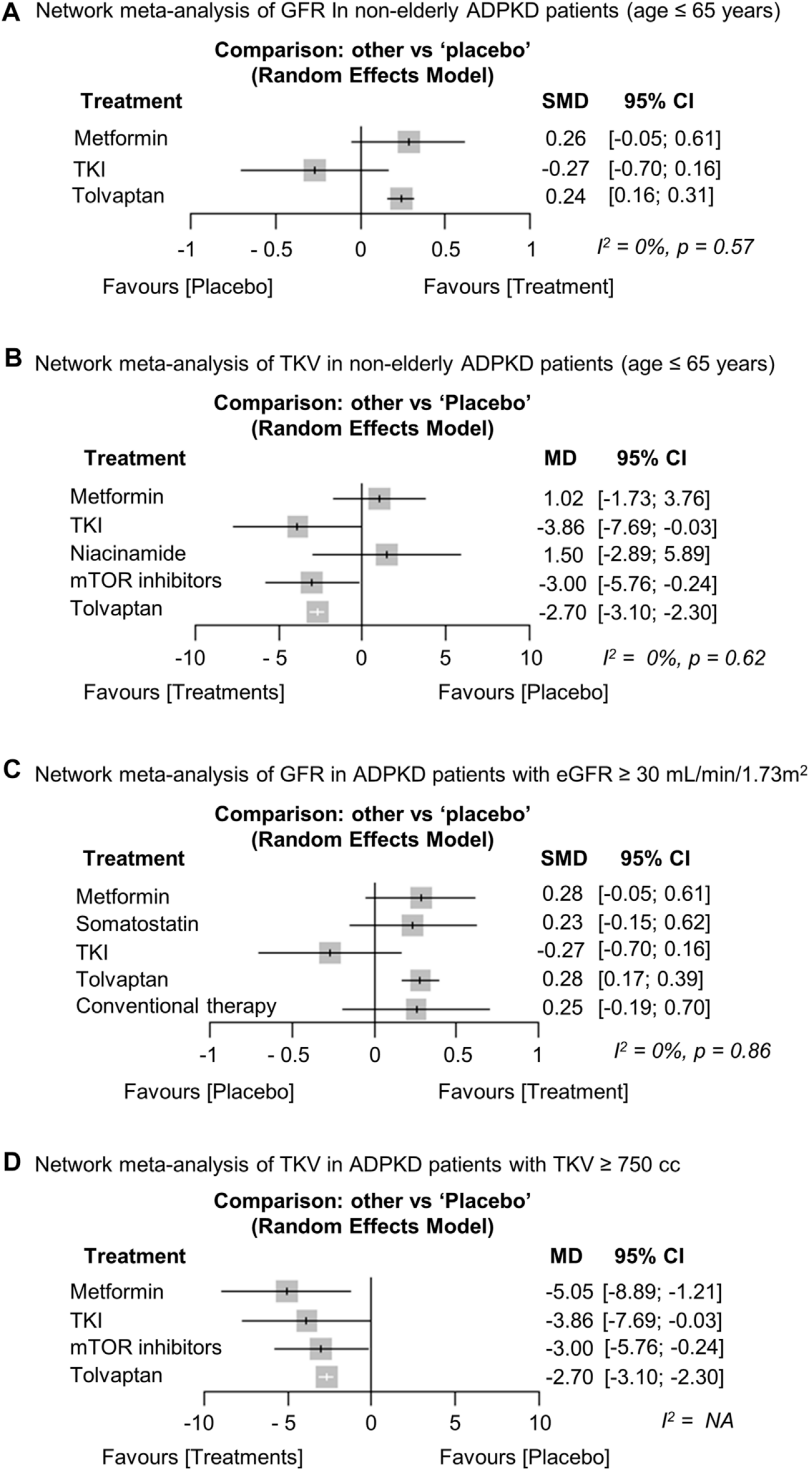
Network meta-analysis regarding the subgroup analysis. Forest plot showing each treatment effect of **(A)** preserving kidney function [glomerular filtration rate (GFR)] and **(B)** inhibiting total kidney volume (TKV) growth rate compared to placebo in non-older adult ADPKD patients (age ≤ 65 years). Forest plot showing each treatment effect of **(C)** preserving kidney function (GFR) in ADPKD patients with baseline eGFR ≥ 30 ml/min/1.73 m^2^ and **(D)** inhibiting TKV growth rate in ADPKD patients with baseline TKV ≥ 750 cc. ADPKD, autosomal dominant polycystic kidney disease; CI, confidence interval; SMD, standard mean difference; MD, mean difference; TKI, tyrosine kinase inhibitor; eGFR, estimated GFR.

We conducted planned subgroup analyses separated by age, baseline eGFR, and TKV. The results for GFR (Torres et al., 2012; Brosnahan et al., 2022; Perrone et al., 2021; Torres et al., 2017a; Tesar et al., 2017) and TKV (Torres et al., 2012; Walz et al., 2010; Brosnahan et al., 2022; Perrone et al., 2021; El Ters et al., 2020; Tesar et al., 2017) were similar to the overall analysis in the subgroup analysis of ADPKD patients ≤65 years (Figures 5A,B). Next, we included metformin, somatostatin analogs, TKIs, and tolvaptan in a subgroup analysis of patients with eGFR ≥30 ml/min/1.73 m^2^. The results showed that only tolvaptan had a superior preserving effect on kidney function compared to the placebo (Torres et al., 2012; Brosnahan et al., 2022; Perrone et al., 2021; Hogan et al., 2020; Meijer et al., 2018; Tesar et al., 2017; Caroli et al., 2013) (Figure 5C), similar to the overall analysis. We also included metformin, TKIs, mTOR inhibitors, and tolvaptan in a subgroup analysis of patients with TKV ≥750 cc. All treatment groups including metformin showed superior efficacy in reducing the TKV growth rate compared to the placebo in the subgroup analysis (Walz et al., 2010; Torres et al., 2012; Tesar et al., 2017; Brosnahan et al., 2022), even though metformin was less effective in the overall analysis (Figure 5D).

We performed an exploratory analysis of the effects of the individual drugs. We did not compare all of the drugs because the network would have disconnected. We analyzed five drugs in the kidney function (GFR) analysis, such as “Metformin,” “Pasireotide,” “Octreotide,” “Bosutinib” and “Tolvaptan” (Torres et al., 2012; Brosnahan et al., 2022; Perrone et al., 2021; Hogan et al., 2020; Perico et al., 2019; Torres et al., 2017b; Tesar et al., 2017; Caroli et al., 2013; Hogan et al., 2010). The results showed that only tolvaptan was significantly more effective in preserving GFR compared to the placebo (Supplementary Figure S5A). Tolvaptan was also statistically superior to bosutinib (TKI), but not significantly different from the other agents (Supplementary Figure S5B). We analyzed seven drugs in the TKV analysis, such as “Metformin,” “Pasireotide,” “Octreotide,” “Niacinamide,” “Bosutinib” “Everolimus,” and “Tolvaptan”(Hogan et al., 2010; Walz et al., 2010; Torres et al., 2012; Caroli et al., 2013; Tesar et al., 2017; Perico et al., 2019; El Ters et al., 2020; Hogan et al., 2020; Perrone et al., 2021; Brosnahan et al., 2022). Pasireotide, octreotide, bosutinib, everolimus, and tolvaptan significantly reduced TKV growth rate compared to the placebo (Supplementary Figure S6A). Moreover, among the somatostatin analogs, only octreotide was significantly better than tolvaptan (Supplementary Figure S6B).

#### 3.3.5 Potential Evidence of Reporting Bias

No evidence of apparent reporting bias was found. The results of the funnel plot, the Egger’s test, and the Begg-Mazumdar test are shown in Supplementary Data (Supplementary Figure S7).

## 4 Discussion

In our meta-analysis, only tolvaptan had significantly greater positive effects on preserving kidney function (GFR) and inhibiting TKV growth compared to the placebo. This result indicates that tolvaptan is a reasonable gold standard treatment for ADPKD. In addition, the somatostatin analogs, TKIs, and mTOR inhibitors significantly suppressed the TKV growth rate compared to the placebo. Surprisingly, somatostatin analogs had a greater TKV growth-suppressive effect than tolvaptan. Octreotide-LAR had a particularly high therapeutic effect among the somatostatin analogs. The results of the other subgroup analyses were mostly similar to the overall results. However, it was interesting that metformin was effective in inhibiting TKV growth in the group with a relatively large TKV (≥ 750 cc).

Vasopressin is an antidiuretic hormone that binds to V2R in the collecting ducts and connecting tubules (Kortenoeven and Fenton, 2014) and activates adenylyl cyclase (AC) via a G-protein, thereby increasing cAMP. Vasopressin signaling contributes to the growth of cysts because, as mentioned before, increasing cAMP promotes cyst growth (Gattone et al., 2003; Sussman et al., 2020). Therefore, tolvaptan blocks the V2R and inhibits cyst growth by reducing intracellular cAMP levels (Torres et al., 2012; Torres et al., 2017a; Sussman et al., 2020). By a similar mechanism, suppressing vasopressin secretion by drinking adequate water also reduces renal cyst growth in several PKD models (Nagao et al., 2006; Hopp et al., 2015). Furthermore, polycystic kidney (PCK) rats lacking circulating vasopressin have remarkably impaired renal cAMP levels and form cysts, but vasopressin treatment completely restores the cyst phenotype (Wang et al., 2008). These results are strong evidence that vasopressin plays an important role in ADPKD and that the inhibitor tolvaptan had a significant effect.

Somatostatin is a hormone secreted by the nerves, gastrointestinal tract, and pancreas (Messchendorp et al., 2020). Somatostatin and somatostatin analogs bind to somatostatin receptors (SSTRs), inhibit AC activity, and reduce cAMP production by maintaining intracellular calcium levels (Messchendorp et al., 2020). In the present study, treatment with somatostatin analogs was effective at inhibiting TKV growth compared to tolvaptan. One of the reasons for this is that V2R is mainly expressed in the distal nephron and collecting duct, whereas SSTRs are widely distributed in the thick ascending loop of Henle, distal tubules, collecting ducts, and proximal tubules; thus, somatostatin analogs inhibit cyst enlargement in these areas (Sussman et al., 2020). In addition, several clinical studies have shown that somatostatin analogs inhibit not only renal cysts but also hepatic cyst growth (Ruggenenti et al., 2005; van Keimpema et al., 2009; Hogan et al., 2012; Pisani et al., 2016). Tolvaptan does not inhibit hepatic cyst growth; SSTRs are expressed in cholangiocytes (Masyuk et al., 2017), which may lead to different effects on cysts.

Metformin is a novel and attractive therapeutic candidate for ADPKD. Metformin inhibits the mTOR and CFTR pathways and activates AMPK, which has been associated with reduced renal cyst growth in an ADPKD mouse model (Takiar et al., 2011; Carullo et al., 2021; Pastor-Soler et al., 2022). In the present analysis, metformin also showed a favorable trend, but it was not significant, possibly because of a small sample size. In addition, the advantage of metformin is that it is already a widely used drug for treating diabetes mellitus. It is expected that further evidence will accumulate in the future for applying metformin as an ADPKD treatment.

mTOR is activated in ADPKD, which is targeted by mTOR inhibitors. Previous meta-analyses did not show positive nephroprotection (Lin et al., 2019), but the present study showed a better effect than the placebo in inhibiting TKV growth.

Interestingly, the conventional therapy group in this analysis was more effective than the placebo group in reducing TKV growth, possibly because of bias due to a lack of complete blinding or the conventional therapy group may have received more strict dietary advice and antihypertensive management than the placebo group. It has been reported that restricting sodium and controlling blood pressure benefit the management of ADPKD (Schrier et al., 2014; Torres et al., 2017b).

AEs were also examined, but no significant differences in serious AEs were observed between the treatments and placebo. Although the major AEs differed between the treatments, there were no AEs referred in this analysis that would substantially limit treatment options.

The main strength of this study is the first network meta-analysis comparing treatments for ADPKD; we showed the validity of using tolvaptan and the therapeutic potential of somatostatin analogs (octreotide-LAR) for ADPKD patients. In addition, our data suggested metformin as a potential new treatment for ADPKD. Another strength of our study is that we were able to analyze more than 4,000 patients, although ADPKD clinical trials often have relatively small sample sizes, and most analyses in this study showed low heterogeneity.

Our meta-analysis had several limitations. First, more than half of the treatment group received the tolvaptan treatment; thus, the sample size for the other treatment groups was relatively small, and some of the patient backgrounds were not consistent. Second, no RCTs were included that directly compared the therapeutic effects of the drugs. Third, in the present study, the outcomes related to the effect of treatment on ADPKD patients were limited: changes in total liver volume (including liver cysts) and subjective symptoms such as quality of life associated with abdominal distention, kidney pain and other ADPKD complications could not been evaluated. Fourth, we were unable to analyze the reasons for the discrepancy between the inhibition of TKV growth and the maintenance of kidney function. Therefore, the results suggested in this analysis that somatostatin analogs were more effective than tolvaptan in inhibiting TKV growth should be interpreted with caution and examined in a direct comparison study. Further studies are also needed to evaluate whether TKV growth suppression improves outcomes other than kidney function (e.g., whether it improves quality of life by relieving symptoms such as kidney pain and abdominal distention).

In the present study, the differences of effects between preserving GFR and inhibiting TKV growth were observed in some drugs. Considering pathophysiology of ADPKD, these should be correlated. This could be attributed to characteristics of included clinical trials. The increases in TKV have been reported to precede the changes in GFR (Torres et al., 2018), which may be another reason for the discrepancy in results. As the progression of ADPKD is variable among individuals but generally slow, studies evaluating these effects ideally need large sample size and long follow-up time (Bergmann et al., 2018). However, such designs of clinical trials are hardly feasible in emerging drugs. We tried to improve the issue through synthesizing results of RCTs using a network meta-analysis, but it has not been completely resolved. Further clinical trials are needed to address this issue. Clinical trials with several drugs included in this study are ongoing. Those studies, such as a direct comparison of the treatment effects of tolvaptan and metformin (NCT03764605), will provide further insight into ADPKD treatments.

In conclusion, only tolvaptan preserved kidney function in ADPKD patients compared to the placebo. Somatostatin analogs, TKIs, mTOR inhibitors, and tolvaptan were effective at inhibiting TKV growth compared to the placebo. Notably, the results suggested that somatostatin analogs were more effective than tolvaptan in inhibiting TKV growth, but need to be validated in further large direct comparative studies.

## Data Availability

The raw data supporting the conclusion of this article will be made available by the authors, without undue reservation.
