# Supplementary material for: Comparative Efficacy of Pharmacological Treatments for Adults With Autosomal Dominant Polycystic Kidney Disease: A Systematic Review and Network Meta-Analysis of Randomized Controlled Trials
Source: Front Pharmacol. 2022 May 18;13:885457. doi: 10.3389/fphar.2022.885457 (PMC9158498; doi:10.3389/fphar.2022.885457)
Supplement: Supplementary file 1 [file DataSheet1.PDF]

Supplementary materials

Table S1. More detailed patient characteristics and baseline parameters

First author - Year	Treatment group	Number of patients (% male)	Race — no. (%)	eGFR (mL/min/ 1.73m^2)	Stratification on kidney function — no. (%)	TKV (mL)	htTKV (mL/m)	Stratification on TKV — no. (%)	Mayo class — no. (%)						BP (mmHg)	Hypertension — no. (%)	RAS inhibitor treatment — no. (%)
									1A	1B	1C	1D	1E	2			
Brosnahan GM -2021	Metformin	26 (42)	Non-Hispanic White: 26 (100)	68	eGFR < 60 mL/min/1.73 m^2 : 6 (23)	2101	1281	-	1 (4)	6 (23)	8 (31)	7 (27)	4 (15)	-	124/80	24 (92)	20 (77)
	Placebo	25 (32)	Non-Hispanic White: 24 (96)	72	eGFR < 60 mL/min/1.73 m^2 : 5 (20)	1156	688	-	2 (8)	7 (28)	14 (56)	1 (4)	1 (4)	-	125/82	19 (76)	13 (52)
Perrone RD -2021	Metformin	49 (-)	-	86	-	-	626	-	9 (18.8)	13 (27.1)	14 (29.2)	6 (12.5)	4 (8.3)	2 (4.2)	122/77	-	-
	Placebo	48 (-)	-	86	-	-	751	-	6 (12.5)	15 (31.3)	12 (25.0)	6 (12.5)	4 (8.3)	5 (10.4)	124/75	-	-
Hogan MC -2020	Pasireotide LAR	33 (20)	-	74	-	-	534	-	-	-	-	-	-	-	-	-	-
	Placebo	15 (6)	-	76	-	-	397	-	-	-	-	-	-	-	-	-	-
El Ters M -2020	Niacinamide	18 (56)	Caucasian, non-Hispanic: 18 (100)	78	-	-	1210	-	0	3 (17)	5 (28)	5 (28)	5 (28)	0	-	18 (100)	16 (89)
	Placebo	18 (33)	Caucasian, non-Hispanic: 18 (100)	68	-	-	1021	-	0	1 (6)	10 (56)	6 (33)	1 (6)	0	-	14 (78)	13 (72)
Perico N -2019	Octreotide LAR	51 (61)	-	27.9 [a]	CKD stage 3b: 20 (39), CKD stage 4: 31 (61)	2338	1344	-	2 (3.9)	2 (3.9)	16 (31.4)	13 (25.5)	15 (29.4)	-	135/82	-	-
	Placebo	49 (53)	-	25.8 [a]	CKD stage 3b: 17 (35), CKD stage 4: 32 (65)	2591	1528	-	1 (2.0)	6 (12.2)	13 (26.5)	13 (26.5)	14 (28.6)	-	132/83	-	-
Meijer E -2018	Lanreotide	153 (46.4)	White: 147 (96.1)	51	CKD stage 2: 41 (26.8), CKD stage 3: 112 (73.2)	2046	1138	-	24 (15.7)			119 (77.8)		6 (3.9)	132/82	-	124 (81.1)
	Conventional therapy	152(46.7)	White: 148 (97.4)	51	CKD stage 2: 41 (27.0), CKD stage 3: 109 (71.7)	1874	1029	-	25 (16.4)			120 (78.9)		5 (3.3)	133/82	-	126 (82.9)
Torres VE -2017	Tolvaptan	683 (50.8)	White: 626 (91.7)	41	CKD stage 2: 32 (4.7), stage 3: 512 (75.0), stage 4: 139 (20.4)	-	-	-	-	-	-	-	-	-	129/82	634 (92.8)	595 (87.1)
	Placebo	687 (48.5)	White: 632 (92.0)	41	CKD stage 2: 39 (5.7), stage 3: 517 (75.6), stage 4: 128 (18.7)	-	-	-	-	-	-	-	-	-	130/83	640 (93.2)	581 (84.6)
Tesar V -2017	Bosutinib	113 (50)	White: 104 (92)	87	-	1393	-	> 1500 mL: 50 (44), 750 – 1500 ml: 64 (56)	-	-	-	-	-	-	-	-	-
	Placebo	56 (37)	White: 53 (95)	87	-	1392	-	> 1500 mL: 25 (45), 750 – 1500 ml: 31 (55)	-	-	-	-	-	-	-	-	-
Ruggenenti P -2016	Sirolimus	21 (42.9)	-	27 [a]	-	2858	-	-	-	-	-	-	-	-	136/86	-	19 (90)
	Conventional therapy	20 (40)	-	31 [a]	-	3123	-	-	-	-	-	-	-	-	134/86	-	19 (95)
Braun WE -2014	Sirolimus	20 (50)	White: 20 (100)	68	Initial iGFR 25–59 ml/min per 1.73 m^2: 7 (35)	2099	-	Initial TKV>1500 mL: 13 (65)	-	-	-	-	-	-	-	6 (30)	-
	Conventional therapy	10 (70)	White: 9 (90)	70	Initial iGFR 25–59 ml/min per 1.73 m^2: 2 (20)	2072	-	Initial TKV>1500 mL: 6 (60)	-	-	-	-	-	-	-	5 (50)	-
Caroli A -2013	Octreotide LAR	40 (42.5)	-	90	-	1557	906	-	-	-	-	-	-	-	127/84	-	-
	Placebo	39 (51.3)	-	76	-	2161	1267	-	-	-	-	-	-	-	127/84	-	-
Torres VE -2012	Tolvaptan	961 (51.5)	White: 810 (84.3)	81	Estimated creatinine clearance < 80mL: 242 (25.2)	1705	979	Total kidney volume < 1000mL: 197 (20.5)	-	-	-	-	-	-	129/83	765 (79.6)	683 (71.1)
	Placebo	484 (51.9)	White: 408 (84.3)	82	Estimated creatinine clearance < 80mL: 130 (26.9)	1668	958	Total kidney volume < 1000mL: 101 (20.9)	-	-	-	-	-	-	128/83	382 (78.9)	350 (72.3)
Walz G -2010	Everolimus	213 (48.8)	White: 207 (97.2)	53	-	2028	-	-	-	-	-	-	-	-	136/88	187 (87.8)	170 (79.8)
	Placebo	216 (53.7)	White: 216 (100)	56	-	1911	-	-	-	-	-	-	-	-	135/88	190 (88.0)	173 (80.1)
Serra AL -2010	Sirolimus	50 (58)	White: 50 (100)	92	CKD stage 1: 29 (58), stage 2: 18 (36), stage 3: 3 (6)	907	-	-	-	-	-	-	-	-	130/84	36 (72)	22 (44)
	Conventional therapy	50 (64)	White: 49 (98)	91	CKD stage 1: 25 (50), stage 2: 24 (48), stage 3: 1 (2)	1003	-	-	-	-	-	-	-	-	130/83	32 (64)	21 (42)
Hogan MC -2010	Octreotide LAR	28 (17.9)	-	70 [b]	-	1143	-	-	-	-	-	-	-	-	122/80	-	-
	Placebo	14 (7.1)	-	71 [b]	-	803	-	-	-	-	-	-	-	-	121/79	-	-
Fassett RG -2010	Pravastatin	29 (41)	-	59	-	-	-	-	-	-	-	-	-	-	133/88	-	23 (79)
	Conventional therapy	20 (35)	-	50	-	-	-	-	-	-	-	-	-	-	134/82	-	15 (75)

eGFR, estimated glomerular filtration rate; TKV, total kidney volume; htTKV, height-adjusted TKV; BP, blood pressure; BMI, body mass index; LAR, long-acting release; RAS, renin-angiotensin system.
a iohexol GFR. b iothalamate GFR.

Supplementary figure

Figure S1

	Random sequence generation (selection bias)	Allocation concealment (selection bias)	Blinding of participants and personnel (performance bias)	Blinding of outcome assessment (detection bias)	Incomplete outcome data (attrition bias)	Selective reporting (reporting bias)	Other bias
1. Brosnahan GM. 2021.	+	+	+	+	?	+	+
2. Perrone RD. 2021. (TAME PKD)	+	+	+	?	+	+	+
3. Hogan MC. 2020.	+	+	+	?	+	+	+
4. El Ters M. 2020. (NIAC-PKD 2)	+	+	+	+	+	?	+
5. Perico N. 2019. (ALADIN 2)	+	+	+	+	?	+	+
6. Meijer E. 2018. (DIPAK 1)	+	−	−	+	+	+	?
7. Torres VE. 2017. (REPRISE)	+	+	+	?	+	+	+
8. Tesar V. 2017.	+	+	+	?	?	?	?
9. Ruggenenti P. 2016. (SIRENA-II)	+	−	−	+	?	+	?
10. Braun WE. 2014.	?	−	−	?	?	+	?
11. Caroli A. 2013. (ALADIN)	+	?	?	?	?	+	?
12. Torres VE. 2012. (TEMPO 3:4)	+	+	+	?	−	+	?
13. Walz G. 2010.	+	+	+	+	−	+	?
14. Serra AL. 2010. (Suisse ADPKD)	+	−	−	+	+	+	?
15. Hogan MC. 2010.	+	+	+	+	?	+	+
16. Fassett RG. 2010.	+	−	−	−	?	?	?

Figure S2

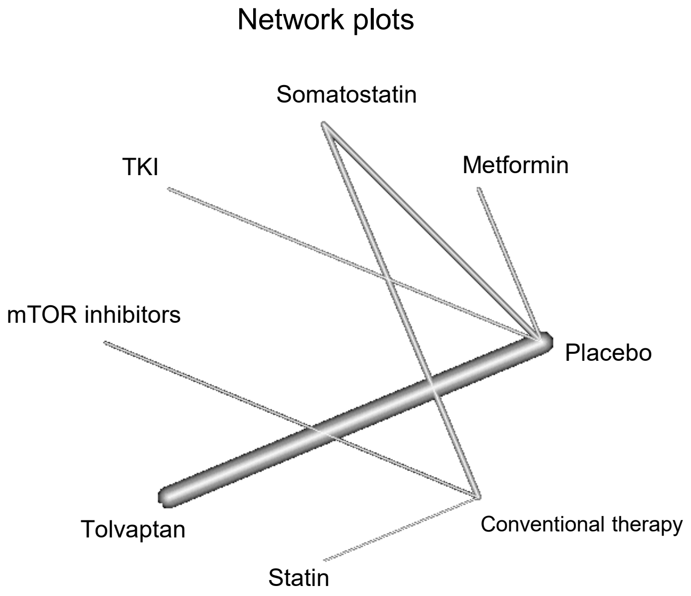

Figure S3

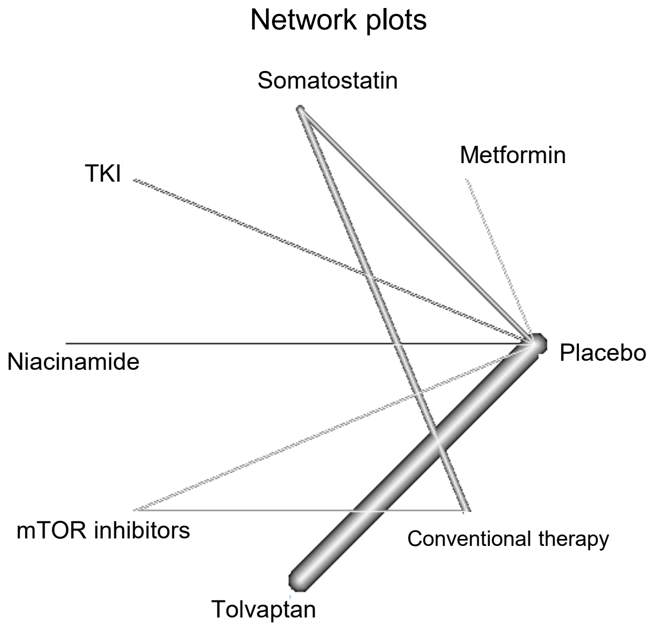

Figure S4

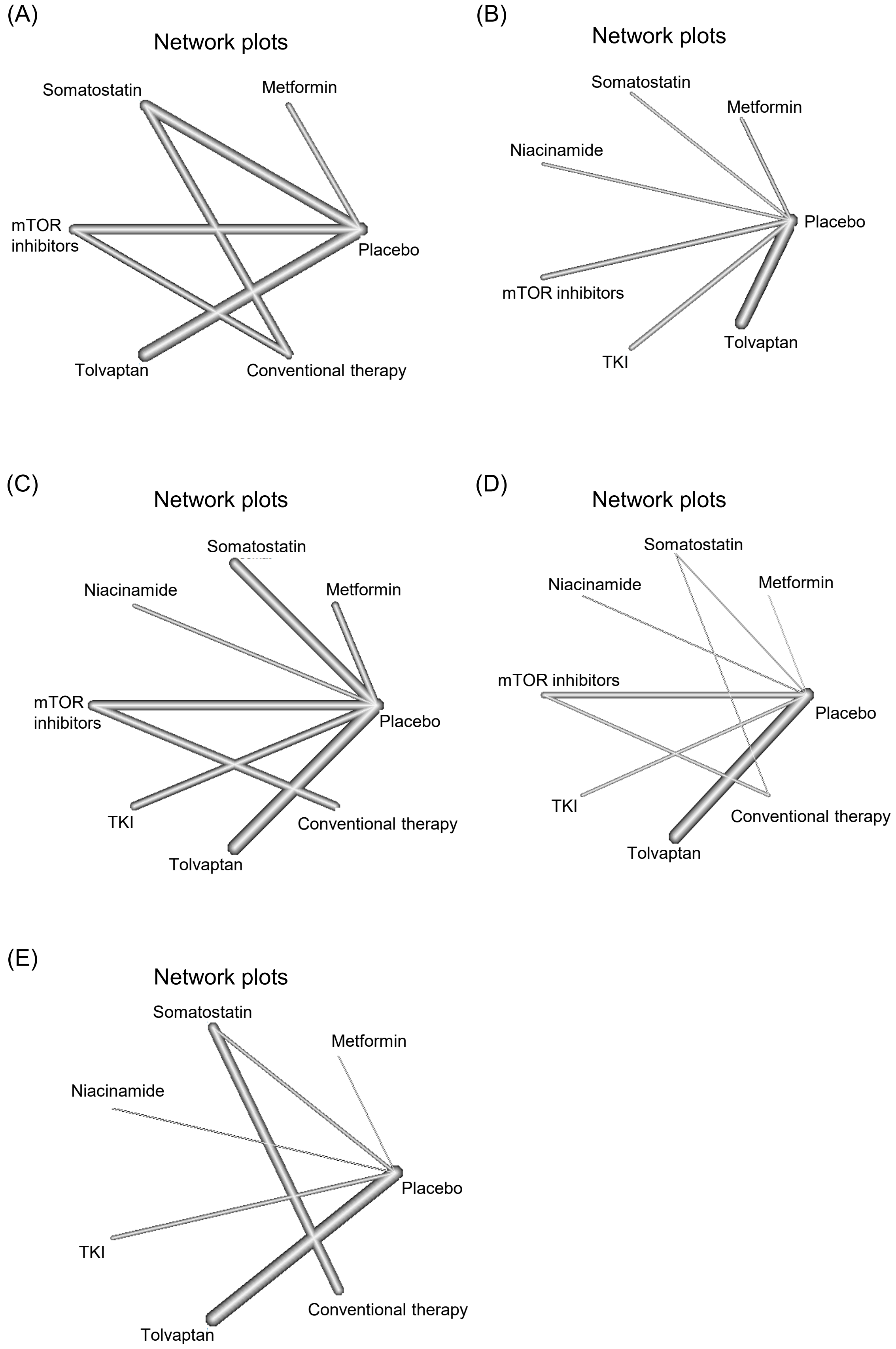

(A)
Network plots
Somatostatin
Metformin
mTOR inhibitors
Placebo
Tolvaptan
Conventional therapy
(B)
Network plots
Somatostatin
Metformin
Niacinamide
Placebo
mTOR inhibitors
TKI
Tolvaptan
(C)
Network plots
Somatostatin
Niacinamide
Metformin
mTOR inhibitors
Placebo
TKI
Conventional therapy
Tolvaptan
(D)
Network plots
Somatostatin
Niacinamide
Metformin
mTOR inhibitors
Placebo
TKI
Conventional therapy
Tolvaptan
(E)
Network plots
Somatostatin
Metformin
Niacinamide
Placebo
TKI
Conventional therapy
Tolvaptan

Figure S5

(A)

(B)

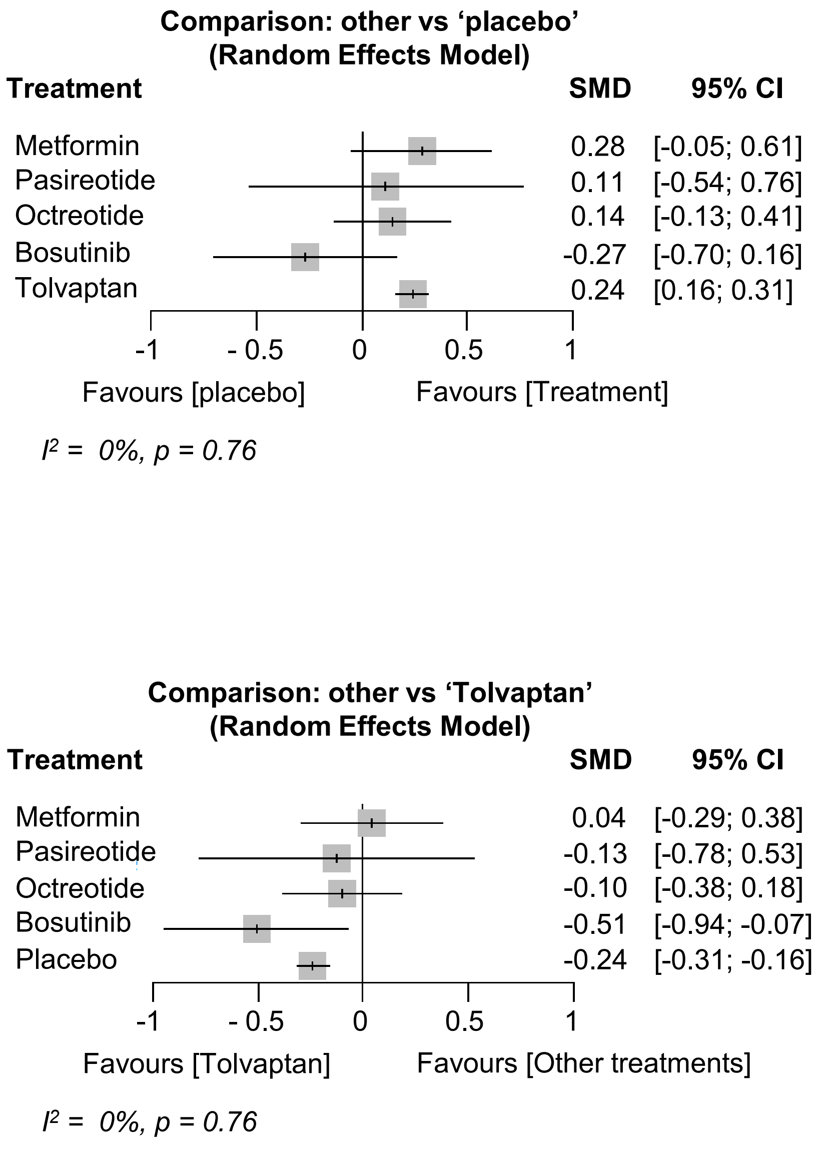

Comparison: other vs ‘placebo’
(Random Effects Model)
Treatment
SMD
95% CI
Metformin 0.28 [-0.05; 0.61]
Pasireotide 0.11 [-0.54; 0.76]
Octreotide 0.14 [-0.13; 0.41]
Bosutinib -0.27 [-0.70; 0.16]
Tolvaptan 0.24 [0.16; 0.31]
-1
- 0.5
0
0.5
1
Favours [placebo]
Favours [Treatment]
I^2 = 0%, p = 0.76
Comparison: other vs ‘Tolvaptan’
(Random Effects Model)
Treatment
SMD
95% CI
Metformin 0.04 [-0.29; 0.38]
Pasireotide -0.13 [-0.78; 0.53]
Octreotide -0.10 [-0.38; 0.18]
Bosutinib -0.51 [-0.94; -0.07]
Placebo -0.24 [-0.31; -0.16]
-1
- 0.5
0
0.5
1
Favours [Tolvaptan]
Favours [Other treatments]
I^2 = 0%, p = 0.76

Figure S6

(A)

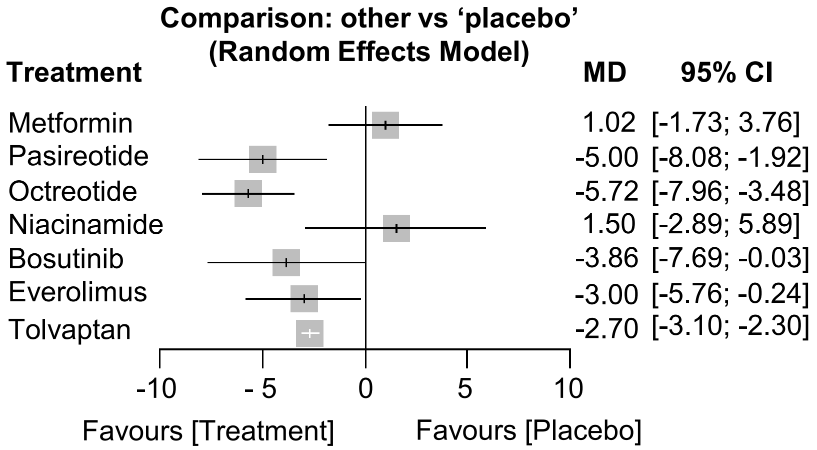

I^2 = 0%, p = 0.60

(B)

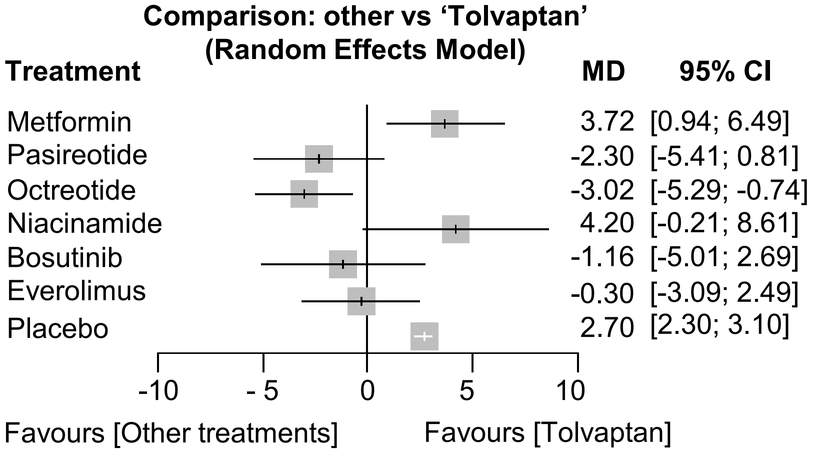

I^2 = 0%, p = 0.60

Figure S7

(A) GFR

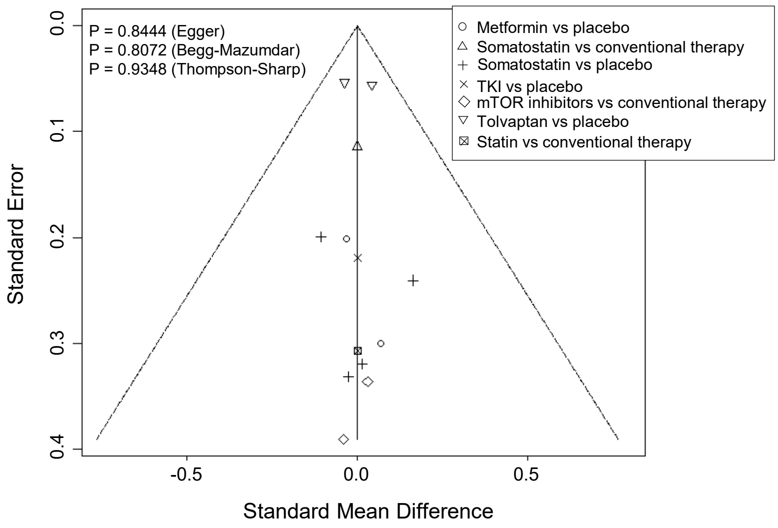

(B) TKV

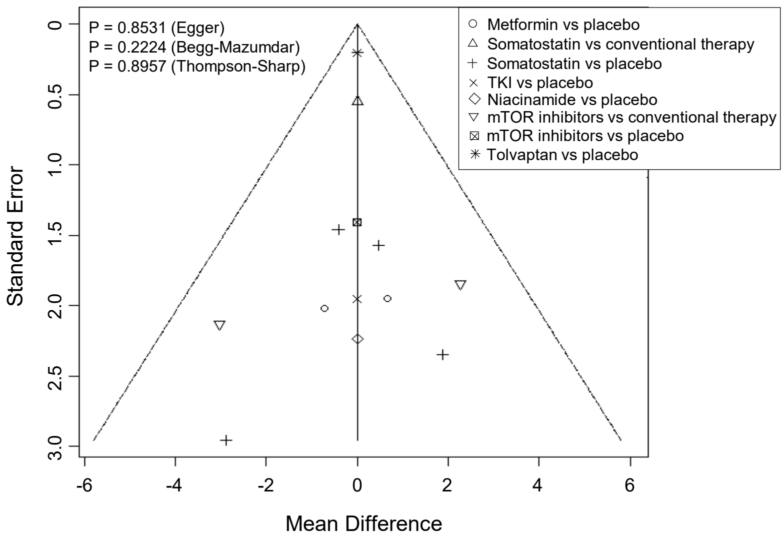

(C) Serious AE

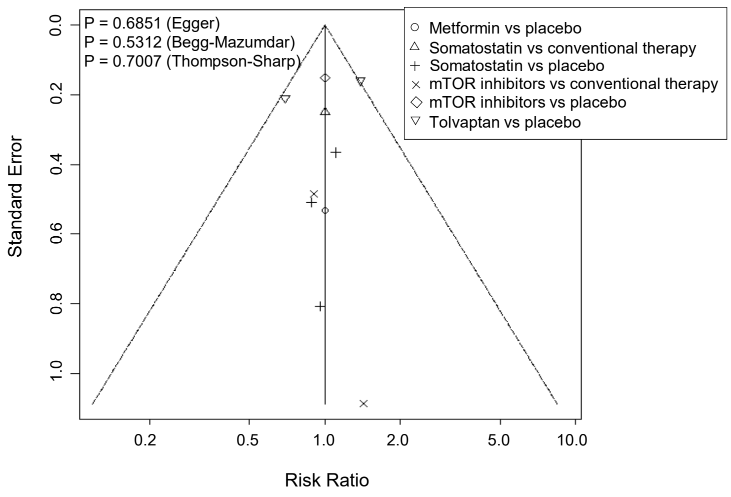

(D) Diarrhea

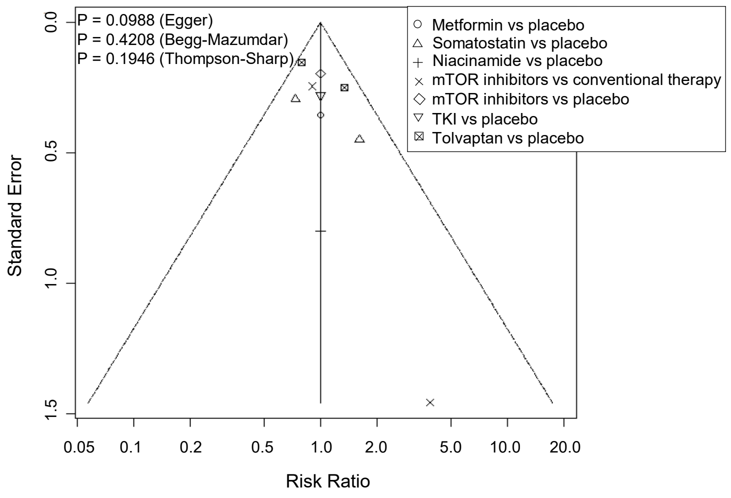

(E) UTI

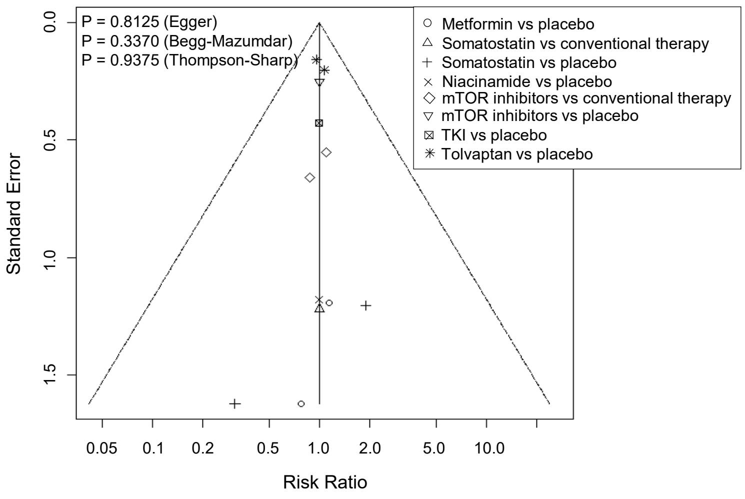

SUPPLEMENTARY FIGURE LEGENDS

Figure S1.

Quality assessment (Cochrane risk of bias tool) for the included RCTs.

RCT, randomized controlled trials

Figure S2.

Network plot for the effect of preserving kidney function.

Figure S3.

Network plot for the effect of inhibiting total kidney volume growth.

Figure S4.

Network plot for the risk ratios (RRs) for adverse events (AEs) regarding (A) serious AEs, (B) nausea/vomiting, (C) diarrhea, (D) urinary tract infection (UTI), and (E) fatigue/weakness.

Figure S5.

Network meta-analysis reporting the standard mean difference (SMD) for each drug effect of preserving kidney function (glomerular filtration rate) compared to (A) the placebo and (B) tolvaptan in ADPKD patients.

CI, confidence interval; ADPKD, autosomal dominant polycystic kidney disease.

Figure S6.

Network meta-analysis reporting the mean difference (MD) for each drug effect of inhibiting total kidney volume (TKV) growth rate compared to (A) the placebo and (B) tolvaptan in ADPKD patients.

CI, confidence interval; ADPKD, autosomal dominant polycystic kidney disease.

Figure S7.

Funnel plot of the effect of each treatment on (A) preserving kidney function and (B) inhibiting total kidney volume (TKV) growth; frequency of (C) serious adverse effects (AEs), (D) diarrhea, and (E) urinary tract infection (UTI).
